# Sickness Behavior in Honey Bees

**DOI:** 10.3389/fphys.2016.00261

**Published:** 2016-06-28

**Authors:** Nadia Kazlauskas, Martín Klappenbach, Amaicha M. Depino, Fernando F. Locatelli

**Affiliations:** ^1^Instituto de Fisiología Biología Molecular y Neurociencias, University of Buenos Aires-CONICETBuenos Aires, Argentina; ^2^Departamento de Fisiología Biología Molecular y Celular, Facultad de Ciencias Exactas y Naturales, University of Buenos AiresBuenos Aires, Argentina

**Keywords:** honey bees, sickness behavior, lipopolysaccharide, locomotion, feeding, social interaction

## Abstract

During an infection, animals suffer several changes in their normal physiology and behavior which may include lethargy, appetite loss, and reduction in grooming and general movements. This set of alterations is known as sickness behavior and although it has been extensively believed to be orchestrated primarily by the immune system, a relevant role for the central nervous system has also been established. The aim of the present work is to develop a simple animal model to allow studying how the immune and the nervous systems interact coordinately during an infection. We administered a bacterial lipopolysaccharide (LPS) into the thorax of honey bees to mimic a bacterial infection, and then we evaluated a set of stereotyped behaviors of the animals that might be indicative of sickness behavior. First, we show that this immune challenge reduces the locomotor activity of the animals in a narrow time window after LPS injection. Furthermore, bees exhibit a loss of appetite 60 and 90 min after injection, but not 15 h later. We also demonstrate that LPS injection reduces spontaneous antennal movements in harnessed animals, which suggests a reduction in the motivational state of the bees. Finally, we show that the LPS injection diminishes the interaction between animals, a crucial behavior in social insects. To our knowledge these results represent the first systematic description of sickness behavior in honey bees and provide important groundwork for the study of the interaction between the immune and the neural systems in an insect model.

## Introduction

When facing an infection, animals suffer several changes in their normal physiology and behavior, which include weakness, lethargy, reduced grooming and general movement, and a loss of appetite and interest in their surroundings (Dantzer, 2001). This change in the motivational state is known as sickness behavior and it is believed to be elicited by a nonspecific, innate immune response, without involvement of the adaptive immune system (Dantzer and Kelley, 1989). A relevant role for the nervous system has also been established, as besides carrying out the physiological defense, the immune and the nervous system can interact generating an immune-induced behavioral response (Hart, 1988). These phenomena underlie a highly organized strategy to cope with an infection, and until now has been shown to be widely conserved across mammal species.

Insects possess nonspecific immune defense mechanisms, with many similarities with vertebrate innate immunity (Vilmos and Kurucz, 1998), but they lack specific acquired immunity. Such simpler however effective immune system provides the basis for discovery of broad immune reactions that might be easily overlooked in vertebrate systems (Chambers and Schneider, 2012). Behavioral changes accompanying molecular immune responses could be determinant when fighting a pathogen, especially in social insects. As individuals live in high-density environments and have frequent physical and social contacts, social insects are more sensitive to infections that could spread rapidly (Cremer et al., 2007). Besides that, group members tend to be genetically similar and thus susceptible to the same pathogen infections. All these characteristics make social insects more prone to propagation of infectious diseases (Cremer et al., 2007), which has been an important selection pressure for the appearance of several strategies to counteract this adverse aspect of group living.

In front of a sick conspecific, social insects can display a broad repertory of strategies to deal with it. On one hand, sick ants improved survival when kept with nest-mates, thanks to grooming and antibiotic secretions (Hughes et al., 2002; Ugelvig and Cremer, 2007). On the other hand, infected ants spent most of their time outside the nest and performed less social interactions with their uninfected counterparts (Heinze and Walter, 2010; Bos et al., 2012). Moreover, forager honey bees with prolonged CO_2_ narcosis abandon their social function and remove themselves from their colonies (Rueppell et al., 2010).

These social responses upon infection can be contradictory, as sick animals can be taken care of or removed from the colony, but they tend to be based on collective actions that will eventually benefit the colony. For example, the self-removal by sick social insects was interpreted as an altruistic behavior and would prevent disease transmission. This phenomenon is known as social immunity (Cremer et al., 2007), and makes social insects particularly interesting for the study of the immune-induced behavioral responses. However, these social responses to sickness could respond to a highly conserved individual response, which alters the behavior of a sick individual and, in consequence, alerts and determines the response of the nest-mates. This kind of response has been mainly studied in mammals as sickness behavior.

Although sickness behavior is a highly conserved response in animals, to our knowledge it has not been systematically studied yet as the coordinated change of several behaviors in insects. In this work we aimed to characterize sickness behavior in the honey bee. We administered a bacterial lipopolysaccharide (LPS) into the thorax of the honey bee *Apis mellifera* to trigger an immune response, and evaluated a set of behaviors previously known to be coordinated under sickness behavior in mammals, such as locomotion, appetite, exploratory behavior, and social interaction. We hypothesize that this individual response is highly conserved, i.e., similar to what has been observed in mammals.

## Materials and methods

### Animals

Honey bees (*A. mellifera)* were obtained from regular hives situated at the Campus of the University of Buenos Aires (34°32′S; 58°60′W). Only pollen foragers were used in all experiments in order to minimize the heterogeneity within the animals' responses. The pollen foragers are easily recognized while they return to the hive with pollen loads on their hind legs. Bees were captured in plastic tubes and carried to the laboratory where they were restrained or treated according to specific procedures. In the laboratory, the bees were kept at room temperature (20–24°C) on a 12:12 h light:dark cycle. Experiments were conducted between 11:00 a.m. and 5:00 p.m. during the whole year, excluding austral winter.

### Drug administration

Injections were performed using a tabulated microcapillary pipette (Sigma-Aldrich). Saline solution was used as vehicle (5 mM KCl, 10 mM NaH_2_PO_4_, pH 7.8) as previously described (Mustard et al., 2010). Bacterial lipopolysaccharide (LPS; *Escherichia coli* LPS, serotype 0111:B4, Sigma-Aldrich, St. Louis, USA) was diluted in vehicle at 1 mg/ml, aliquoted and conserved at −20°C until used. In all cases, 4 μl of saline or LPS were injected into the thorax of the bees (Felsenberg et al., 2011).

### Behavioral testing and statistical analysis

#### Locomotor activity

Animals were collected on the same day of the experiment and they were anesthetized by cooling them shortly on ice. Immediately after receiving a saline or LPS injection, bees were placed individually in a closed 15 cm diameter Petri dish. An Eppendorf tube cap filled with 100 μl of 1M sucrose solution was placed in the middle of each Petri dish, allowing animals to eat *ad libitum* during the experiment. Animals remained in the Petri dish for the 135 min that lasted the observations.

Ten minutes were given to the bees to recover from the anesthesia before starting the test. After that, and every 20 min, a measurement was performed: bees were recorded during 5 min and their position was tracked using a video tracking software (ANY-maze®, Stoelting CO, USA). We measured the distance covered by each bee in each trial and the number of immobility events, taken as the number of times in which the bee stopped walking for longer than one second. Additionally, we evaluated the amount of sucrose solution consumed during the whole test by measuring the remaining solution in the Eppendorf tube cap at the end of the test. Evaporation at the end of the 135 min was evaluated by measuring the remaining solution in a Petri dish with no bee. As the evaporation was not significant compared to the initial 100 μl, it was not taken into account.

We performed a two-way repeated measures analysis of variance (ANOVA) to analyze the distance covered by bees in both groups. As the interaction between factors (trial; group) was significant, we performed two-tailed unpaired Student's *t*-test for each trial, to evaluate the drug effect. We also performed two-way repeated measures ANOVA in order to compare the immobility events in both experimental groups, and as we found a significant interaction between factors we then performed unpaired Student's *t*-test for each trial. Finally, we performed a two-tailed unpaired Student's *t*-test in order to compare the sucrose ingested by the animals in both groups. Results are based on data collected from four independent repetitions of the experiment.

#### Feeding experiment

Animals were collected the day before the experiment and restrained on individual harnesses that allowed movements of antennae and mouthparts. They were fed *ad libitum* the night before the experiment with 1M sucrose solution, in order to equalize their initial nutritional state. After 18–20 h of starvation, animals were touched in their antennae with a droplet of 1M sucrose solution. Bees that did not show a rapid and conspicuous proboscis extension were discarded from the experiment (< 5% of the animals). After that, each animal received an injection of saline or LPS. The bees were further divided in three groups aimed at conducting feeding assays: 60, 90 min or 15 h after the saline or LPS injection. In the last case, since the 15 h test fell within the next day of the injection, all animals were fed until satiation the night between the injection and the feeding test. We did this as otherwise the animals would not survive the long period between the initial feeding (day of capture) and the feeding test (2 days after capture). The assay consisted in feeding the animals with 1 μl droplets of 1M sucrose solution using a micrometer syringe (Gilmont GS-1200). Bees received one droplet after the other until sucrose did not elicit proboscis extension. We measured the total sucrose intake of each bee until this point. Since the average intake value may fluctuate between different batches of bees and different days, every time that the experiments was repeated, saline and LPS-injected bees were run in parallel and the amount of sucrose ingested by each bee was normalized to the average amount ingested by the respective saline injected group. Statistical analysis of differences in food intake in saline- and LPS-injected bees was based on two-tailed unpaired Student's *t*-test.

#### Metabolic rate measurement

Animals were captured on the same day of the experiment and were harnessed as explained above. Before the experiment, they were fed until satiation with 1M sucrose solution. One hour after feeding animals were injected with saline solution or LPS and immediately enclosed into a 250 ml plastic bottle. CO_2_ in the bottle was measured using a respirometer (PS-2002 Xplorer GLXTM, Pasco®). Measurements started 10 min after introducing the bee into the bottle and were repeated every 10 min during 2 h. CO_2_ production for a given interval was calculated as the CO_2_ determined in one measurement minus the CO_2_ determined in the previous measurement 10 min before. Thus, the first period reported in the **Figure 3** correspond to the CO_2_ produced from 10 to 20 min after injection. A two-way repeated measures ANOVA was performed to compare CO_2_ production in both groups along measurements.

#### Antennal movements

Animals were captured on the same day of the experiment and they were harnessed as explained above. Before the experiment, they were fed until satiation and the tip of the right antenna was marked with a small dot of commercial fluorescent green paint. The test was conducted in a dark room and bees were focally illuminated using a 380 nm UV lamp. The paint generated a conspicuous fluorescent dot that allowed us to track and quantify the antennal movements with the ANY-maze software (Stoelting). As bees were already harnessed at the moment of the injection, there was no need to anesthetize them on ice for injection. This allowed us to start the behavioral measurements sooner than in other experiments. The assay started 5 min after injection, and animals were recorded during 7 periods of 5 min every 20 min. A two-way repeated measures ANOVA was performed to analyze antennal movement in both groups along successive measurements.

#### Social interaction experiment

Animals were collected on the same day of the experiment. They were anesthetized by cooling them shortly on ice and then received saline or LPS injection. Immediately after, two nest-mates bees were placed in a closed 15 cm diameter Petri dish with a plastic divisor in the middle that maintained the animals separated from each other. Each compartment had an Eppendorf tube cap filled with 100 μl of 1M sucrose solution that served as feeder. After 1 h the plastic divisor was removed, allowing animals to interact during 10 min, while we video-recorded their activity. Three different kinds of honey bee pairs were formed depending on the injection that each bee had received: SAL-SAL, SAL-LPS, and LPS-LPS. We identified seven different behaviors and divided them into two main categories: (1) social behaviors, i.e., behaviors that involved a clear physical interaction among the two bees: antennal contact, body contact, proboscis extension, and attack; and (2) non-social behaviors, i.e., behaviors in which animals were not interacting: fanning, self-grooming, and walking or being still. A white dot painted on top of thorax of one of the bees was used to identify them. A blind observer quantified the time spent performing each behavior by each bee in the pair.

For analysis, we organized the data in four experimental groups to evaluate the interaction between a SAL or LPS injected bee and a SAL or LPS injected bee, and analyzed the number of events and the time dedicated to each behavior by performing a two-way ANOVA, factor1: treatment of the observed bee; factor2: treatment of the counterpart bee. The analysis was followed by Tukey's multiple comparisons test. In the analysis of the number of proboscis extension we discarded an animal from the LPS-SAL group after being indicated outlier using the ROUT method, with Q = 1% (Motulsky and Brown, 2006).

## Results

### LPS reduces locomotor activity and food intake

Sickness behavior is characterized in many species by a change in a set of different behaviors, including locomotion and feeding. Considering this, our first experiment was to evaluate if the injection of the inflammatory molecule LPS affects these behaviors in honey bees. LPS is a lipopolysaccharide found in the outer membrane of gram-negative bacteria that is known to elicit the immune response in honey bees and other insects (Sluss et al., 1996; Boutros et al., 2002; Altincicek et al., 2008; Richard et al., 2008). Notice that LPS injection is used to trigger the immune response without producing a real infection.

**Figure 1:**
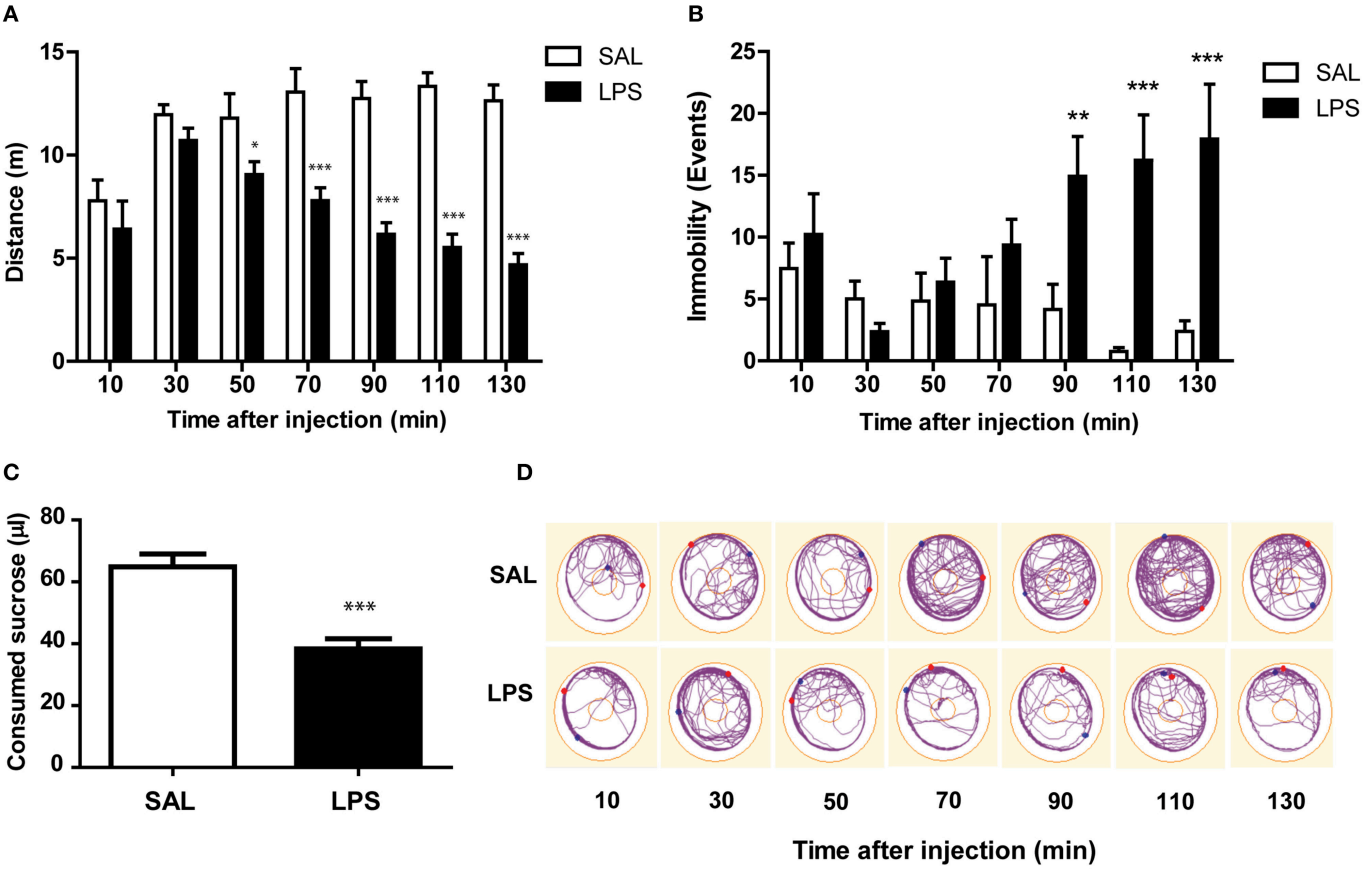
**LPS induces a reduction in locomotor activity and appetite. (A)** Mean and SEM of the distance walked during each trial by bees in each treatment group. **(B)** Mean and SEM of the number of immobility events during each trial by bees in each treatment group. **(C)** Mean and SEM of the amount of sucrose ingested by bees in each treatment group measured at the end of the experiment. **(D)** Representative walking tracks of two bees injected with saline or LPS. Dots correspond to the position of the honeybee at the beginning and the end of the trial. The outer circle represents the arena limit, and the inner circle represents the area occupied by the eppendorf cap that contained sucrose solution. Student's *t*-test: ^*^p < 0.05, ^**^p < 0.01, ^***^p < 0.001. Data from Panels **(A–C)** correspond to the same individuals: N, 13 saline, and 14 LPS-injected bees.

As observed in Figure 1, bees have partially recovered from chill anesthesia 10 min after starting the test, and reach stable locomotion activity 30 min after anesthesia. This level of activity is sustained by saline-injected bees throughout the whole test, whereas LPS-injected bees start showing reduced locomotion in comparison to saline-injected bees 50 min after injection. We performed a repeated measures ANOVA in order to compare the performance of both experimental groups, and found a significant interaction between factors [*F*_(6, 150)_ = 6.36; *p* < 0.001]. Therefore, we compared the effect of the injection at each time point using a two-tailed unpaired Student's *t*-test and found that LPS reduces locomotor activity from 50 min to, at least, 135 min after the injection (Figure 1A). Representative tracks of locomotor activity in saline and LPS-injected animals are shown in Figure 1D.

The decrease in the locomotor activity measured as reduction in accumulated distance could be explained by two different phenomena. On the one hand, LPS-injected bees could be spending the same time walking than saline bees, but doing it more slowly. On the other hand, LPS-injected bees could be spending less time walking and more time being still. To discern between these two possibilities we measured the number of events in which animals stopped their activity at least for 1 s (Figure 1B). We performed a repeated measures ANOVA in order to compare the number of immobility events in both experimental groups, and found a significant interaction between factors [*F*_(6, 150)_ = 4.42; *p* < 0.001]. Thus, we compared the effect of the injection at each time point using a two-tailed unpaired Student's *t*-test and found that LPS administration increases the number of immobility events from 90 min to, at least, 135 min after the injection. Therefore, we conclude that LPS-injected bees spend more time immobile.

At the end of the experiment, we measured the amount of sucrose consumed from the feeder inside the Petri dish. We observed that LPS-injected animals ingested significantly less sucrose solution during the experiment than saline-injected animals [*t*_(25)_ = 4.96; *p* < 0.001; Figure 1C].

These results show that LPS reduces locomotor activity and food intake in honey bees, as it does in mammalian sickness behavior models.

### LPS decreases the amount of sucrose consumed by restrained animals

**Figure 2:**
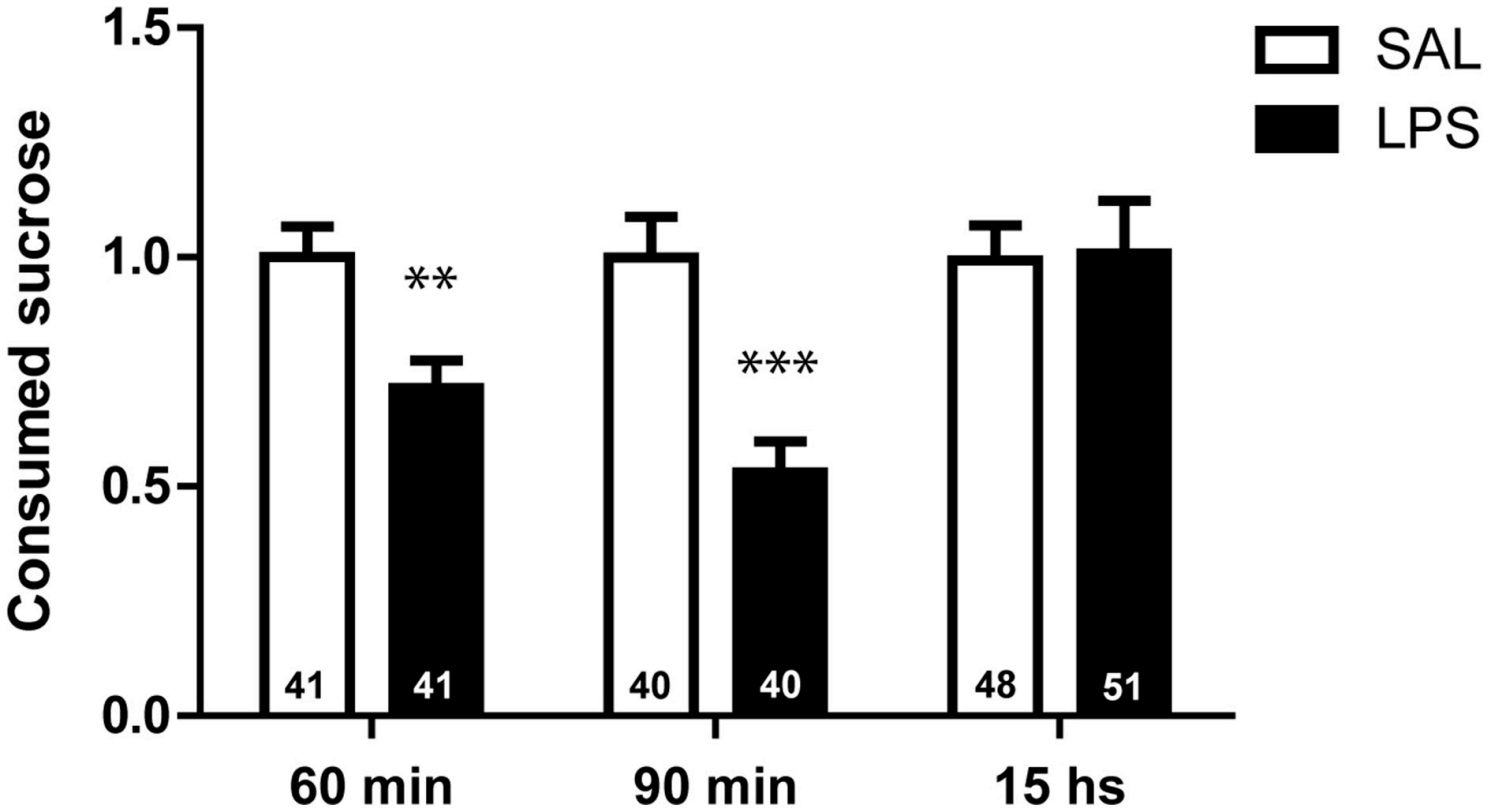
**LPS decreases the amount of sucrose consumed by harnessed animals**. Mean and SEM of the volume of sucrose ingested by bees in each treatment group at different times after injection. 60, 90 min, and 15 h correspond to three independent groups of bees (each bee was measured only once). The volume ingested by each individual bee was normalized to the average volume consumed by bees in the respective saline group. Number of animals is indicated within each bar. Two-tailed unpaired Student's *t*-test: ^**^p < 0.01, ^***^p < 0.001.

The reduction in sucrose intake observed in LPS-injected bees (Figure 1C) could be consequence of the diminished locomotor activity and lower energy demand (Figure 1A), or alternatively it could be due to a loss of appetite as part of the behavioral changes elicited during the sickness behavior (Asarian and Langhans, 2010). To discriminate between these two possibilities, we performed an experiment in which we compared eagerness for food in saline and LPS-injected bees that were physically restrained. The average amount of sucrose consumed by a harnessed bee in the current experimental condition (15–18 h after fed to satiation) was 13.12 ± 0.72 μl, which is notably lower than in the case of bees walking inside the Petri dish (see Figure 1C). In addition, we found that LPS decreases the amount of sucrose consumed by harnessed bees 60 and 90 min after injection [*t*_(80)_ = 3.16; *p* < 0.01 and *t*_(78)_ = 4.23; *p* < 0.001, respectively; Figure 2]. Nevertheless, 15 h later, the amount of sucrose ingested by the LPS and the saline-injected animals were indistinguishable [*t*_(97)_ = 0.10; *p* = 0.92]. In summary, this experiment demonstrates that, as in mammals, injection of LPS diminishes food intake in a reversible manner, i.e., the change observed in LPS-injected bees was not the consequence of a severe injury.

### LPS does not alter the metabolic rate

**Figure 3:**
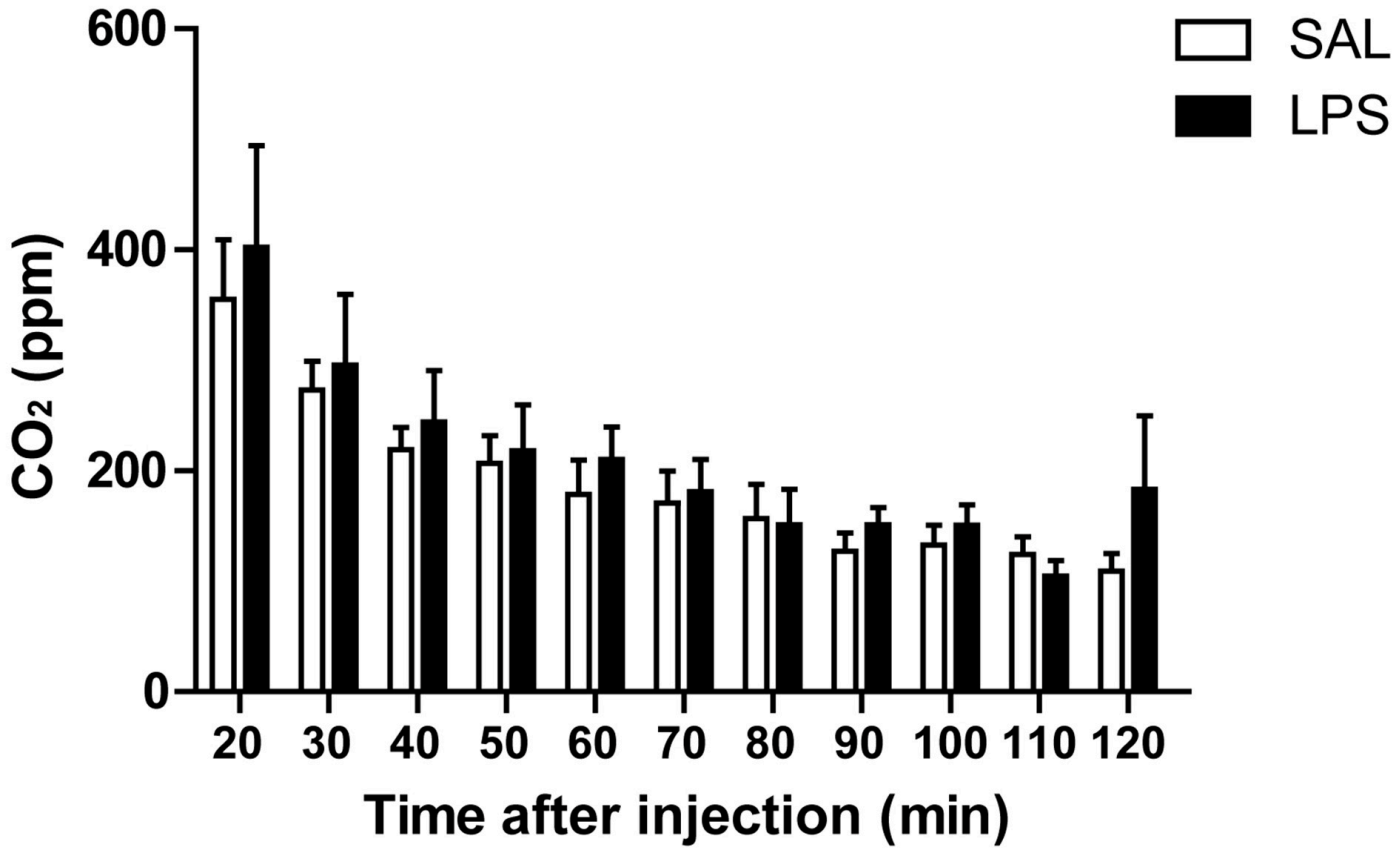
**LPS does not affect the metabolism of honey bees**. Mean and SEM of CO_2_ production along the experiment by bees in each treatment group. N = 11 in saline and in LPS-injected groups.

We have established that LPS decreases sucrose consumption in bees. This effect might correspond, as in other models, to a loss of appetite. However, we also considered that such reduction in food intake might reflect a modulation in the metabolic rate as a symptom of the LPS injection and as part of the strategy to cope with infection. We performed an experiment in order to evaluate whether LPS injection affects the metabolic rate during the time that we have measured changes in food intake and locomotion. Metabolic rate of the animals was assessed by measuring CO_2_ production every 10 min and for 2 h after LPS or saline injection (Figure 3). Repeated measures ANOVA showed no significant interaction between factors [*F*_(10, 200)_ = 0.36; *p* = 0.96]. Therefore, we analyzed the main factors, and found no differences between the two groups [group factor: *F*_(1, 19)_ = 0.002; *p* = 0.97], which indicates that LPS does not change the bee's metabolic rate. Interestingly, the metabolic rate decreases over time in both groups [time factor: *F*_(10, 200)_ = 14.1; *p* < 0.001].

### LPS reduces spontaneous antennal movements

**Figure 4:**
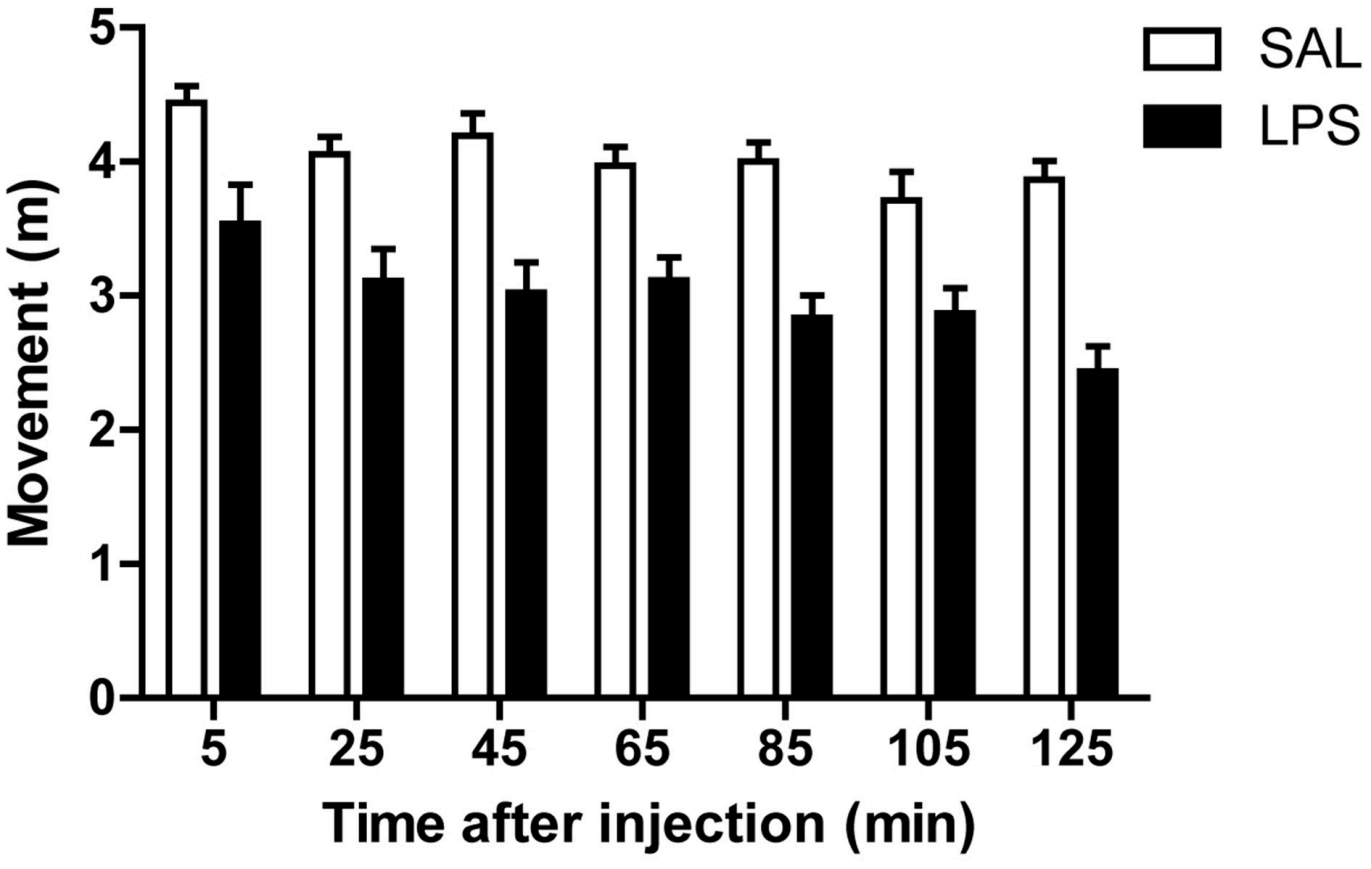
**LPS reduces spontaneous antennal movements**. Mean and SEM of the cumulative antennal movements in each trial along the experiment by bees in each treatment group. N, 11 saline, and 12 LPS-injected animals.

Bee's antennae house receptors for several sensory modalities, including chemical, mechanical, thermal, and hygroreception (Whitehead and Larsen, 1976; Ai et al., 2007). Using antennal scanning of objects, bees can discriminate between shapes, textures, and sizes and can associate them with rewards (Kevan and Lane, 1985; Erber et al., 1998; Scheiner et al., 2001). Moreover, the speed and directionality of the antennal movements change in front of appetitively conditioned odors (Cholé et al., 2015). Therefore, antennal movements are a good indicator of exploratory behavior. We evaluated antennal movements in harnessed animals found no significant interaction between treatment and time [*F*_(6, 120)_ = 1.60; *p* = 0.15]. Therefore, we analyzed the main factors, and found that LPS-injected bees showed an immediate reduction in the antennal movements that persisted throughout the whole test [group factor: *F*_(1, 20)_ = 22.44; *p* < 0.001; Figure 4]. Also, we found a significant decrease in the movements along the experiment for both groups, which might be explained by habituation to the experimental context [time factor: *F*_(6, 120)_ = 9.55; *p* < 0.001].

### LPS decreases social interaction between bees

Animals living in societies have different responses in front of sick conspecifics: they can help the sick individual or, on the contrary, they can remove it, isolating it from the group. Moreover, self-removal from the colony has also been documented. All these behaviors could represent adaptive advantages and they have been found to occur in nature (Arathi et al., 2000; Ugelvig and Cremer, 2007; Rueppell et al., 2010; reviewed in Cremer et al., 2007). As it was also established that sociability decreases as a feature of sickness behavior in different species (Dantzer and Kelley, 2007), we aimed to evaluate LPS effects on social behavior in honey bees.

Three kinds of honey bee pairs were formed depending on the injection they had received: SAL-SAL; SAL-LPS, and LPS-LPS. We evaluated whether the social behavior displayed by LPS-injected bees differed from the social behavior of saline-injected bees. In addition, we assessed whether the social behavior toward LPS-injected bees differs from the social behavior toward saline-injected bees.

**Figure 5:**
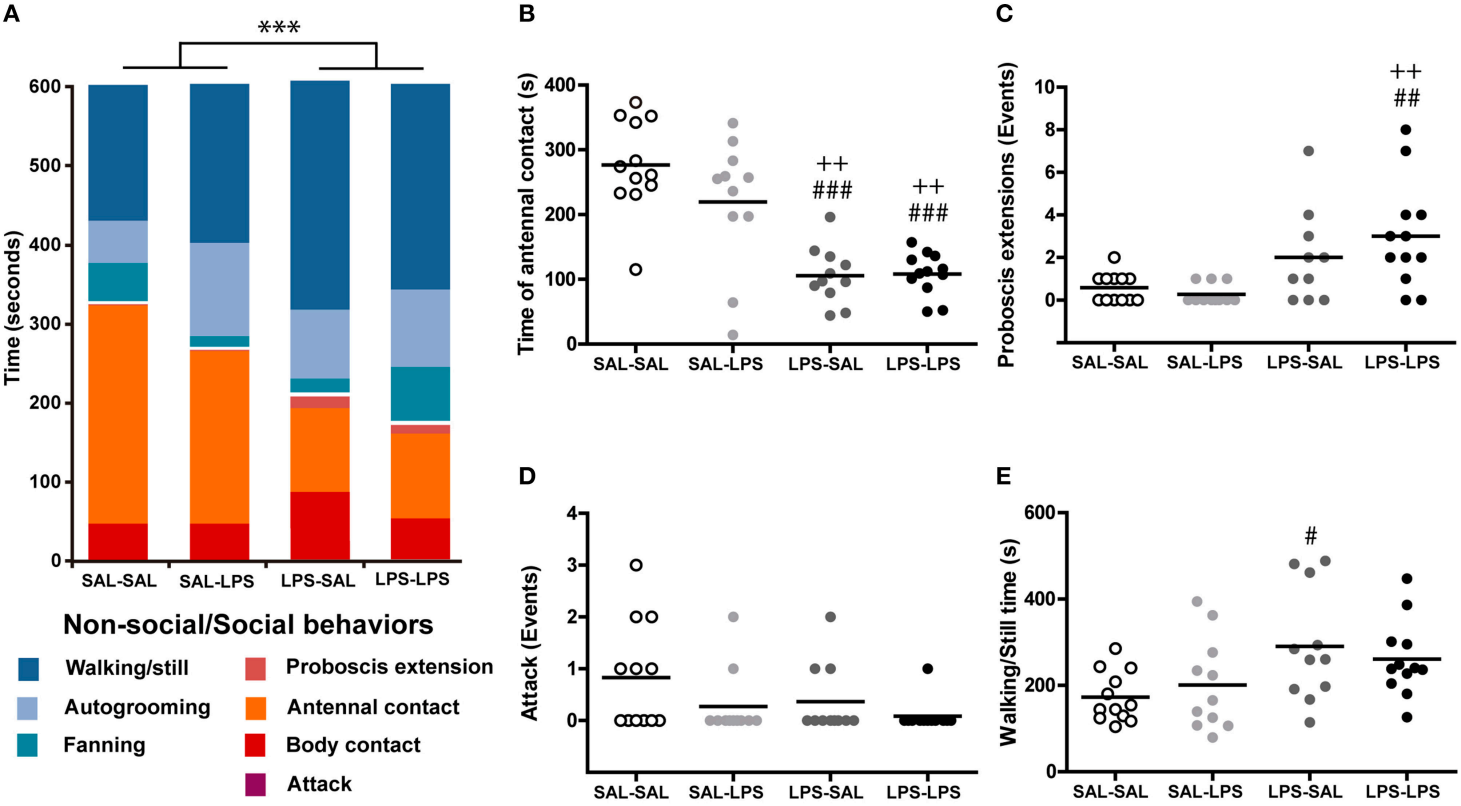
**LPS decreases social interaction between bees. (A)** Ethogram of bees in the different treatment groups shown as fractions of time spent in different behaviors. Blue fractions correspond to non-social behaviors while red-orange fractions correspond to the social behaviors indicated in the figure. Total time spent in attacks is indistinguishable at this time scale. **(B)** Time spent in antennal contact by each animal in the different groups. Events of **(C)** proboscis extension and **(D)** attacks carried out by each animal in each group. **(E)** Time spent in walking or being still by each animal in each group. N, SAL-SAL = 12, SAL-LPS = 11, LPS-SAL = 11, LPS-LPS = 12, except for LPS-SAL group in graph **(C)** where N = 10. Each circle stands for a single animal. Horizontal bars represent the mean of the group. Two-way repeated measures ANOVA ^***^p < 0.001. *Post-hoc* Tukey test comparisons against SAL-SAL group, #*p* < 0.05, ##*p* < 0.01, ###*p* < 0.001; comparisons against SAL-LPS group (++) *p* < 0.01.

We performed a two-way ANOVA in order to compare the time spent performing social behaviors and found that LPS-injected bees dedicated less time to social behaviors regardless of the treatment of their counterpart [group factor: *F*_(1, 42)_ = 18.5; *p* < 0.001, counterpart factor: *F*_(1, 42)_ = 3.71; *p* = 0.06, interaction: *F*_(1, 42)_ = 0.18; *p* = 0.67; Figure 5A]. The differences resulted mainly from differences in the time invested in doing antennal contacts, as this was the most prevalent social behavior that we observed (Figure 5B). Interestingly, the time dedicated to antennal contact was influenced by the treatment of the bee executing it but not by the treatment of the counterpart bee, which might indicate that LPS injection does not elicit noticeable signals to other bees at least 1 h after injection [Two-way ANOVA, group factor: *F*_(1, 42)_ = 51.3; *p* < 0.001; counterpart factor: *F*_(1, 42)_ = 1.88; *p* = 0.18, interaction: *F*_(1, 42)_ = 2.29; *p* = 0.14; Figure 5B].

In addition, LPS-injected bees were found to extend their proboscis toward their partners more often than saline-injected bees [Two-way ANOVA, group factor: *F*_(1, 41)_ = 16.5; *p* < 0.001, counterpart factor: *F*_(1, 41)_ = 0.46; *p* = 0.50, interaction *F*_(1, 41)_ = 1.65; *p* = 0.21; Figure 5C]. This behavior has been normally considered as a food begging display (Wright et al., 2012); however, in all these situations we have not observed any trophallaxis from the partner bee, irrespective of its treatment.

As for the aggressive behavior, we found no significant differences between the number of attack events shown by saline and LPS-injected bees [Two-way ANOVA, group factor *F*_(1, 42)_ = 2.46; *p* = 0.12, counterpart factor: *F*_(1, 42)_ = 4.00; *p* = 0.052, interaction: *F*_(1, 42)_ = 0.44; *p* = 0.51; Figure 5D]. However, for this parameter, there was a tendency to less attack events toward LPS-injected bees, suggesting that sick bees may suppress behaviors that incite aggressiveness from their partner bees.

In regards to time invested in non-social behaviors, we found that LPS-injected bees dedicated more time walking or being still, ignoring the partner in the Petri dish [group factor: *F*_(1, 42)_ = 9.34; *p* < 0.01, counterpart factor: *F*_(1, 42)_ = 0.01; *p* = 0.98, interaction: *F*_(1, 42)_ = 1.00; *p* = 0.32; Figure 5E].

Notice that even though more behavioral patterns are described in the literature, they are not reported here because we have not observed them in our experiment. Taken together, all these results demonstrate that LPS changes the patterns of social behavior and decreases sociability in honey bees, as it has been reported in other sickness behavior models.

## Discussion

With the aim of establishing an insect model that might be used as a simpler system to study the interaction between the nervous and the immune systems, we show here that the administration of the inflammatory molecule LPS, a lipopolysaccharide found in the outer membrane of bacteria, affects a set of behaviors that in other animal models have been shown to be modulated during sickness as part of an orchestrated innate immune response. It is relevant in this analysis, that we have not injected the bees with any agent or pathogen that produces an actual illness or infection, rather a molecule that signals a potential infection and elicits the innate immune response (Moret and Schmid-Hempel, 2000; Mallon et al., 2003; Korner and Schmid-Hempel, 2004; Richard et al., 2008). Thus, all the measured changes are not symptoms related with the progress of an illness, they are rather generated as part of the innate response to face it. Consistent with this idea we show in Figure 2, that the effect of LPS on food intake vanishes 15 h after injection and we have not observed any increased death rate until that time. This observation is consistent with a previous study which shows that LPS administration does not affect bee's survival (Köhler et al., 2012).

We observed that LPS-injected bees have reduced locomotor activity, measured as a shorter distance walked inside a Petri dish (Figure 1A). The decrease in total activity is mainly explained by animals spending more time being still, and not due to decreased walking velocity (Figure 1B). The reduction in movements caused by LPS was also accompanied by a reduction in spontaneous antennal movements (Figure 4). Since honey bees continuously move their antennae to actively sense the environment, the reduction in antennal movements together with lower walking activity, suggest a reduced motivation to explore the surroundings. These results are consistent with a state of lethargy described as part of sickness behavior in mammals (Dantzer and Kelley, 2007).

We also demonstrate that LPS administration reduces food intake in freely walking bees (Figure 1C). The fact that this result was repeated in harnessed bees with restricted movements (Figure 2) indicates that the lower food intake in LPS-injected bees is not mere consequence of the less intensive walking and consequent lower energy consumption. In agreement with this interpretation are the results showing no effect of LPS on the metabolic rate (Figure 3). We found that the metabolic rate decreases over time in both groups, probably explained by the initial stress, which slowly fades as animals get habituated to the experimental situation; however, no change specific to LPS injection was observed. This finding contrasts with results from other studies, which reported lower metabolic rate during infection in two insects (Gray and Bradley, 2006; Arnold et al., 2013). However, in those cases the change in metabolic rate was found days after inoculation with actual pathogens and during advanced infections. In our metabolic rate measurement, we cannot discard that the development of the immune response demands energy, which is compensated by the lower motor activity. In that case we have to assume that changes in food intake must be part of the behavioral strategy to optimize metabolic resources. As central foragers that collect food for the colony, worker honey bees normally ingest amounts of food that constitute more than their individual requirement. In this context it can be considered that during sickness, bees can reduce their food intake without compromising its survival as long as they do not have to share food with their nest mates. All together our results indicate that feeding behavior is actively modulated during sickness, what is interpreted in other models as an induced loss of appetite (Dantzer, 2001).

As changes in food intake cannot be explained solely by the reduction in motor activity, neither changes in motor activity can be explained by diminished food intake. This interpretation arises from the experiments in which antennal movements were diminished in LPS-injected bees, as these animals had been fed until satiation minutes before LPS or saline injection (Figure 4).

In mammals, anorexia has been very well characterized in response to illness and it is proposed to help animals to face infections (Poon et al., 2015). However, for invertebrates there are contradictory results regarding this issue. First, in accordance with the bibliography for vertebrates, it was found that LPS produces anorexia in the locust (Goldsworthy, 2010). Second, the opposite effect has been reported in bumblebees, as they increase their food intake (Tyler et al., 2006). Finally, in honey bees, sucrose intake was measured for an extended period of a week after LPS injection and no change was found (Köhler et al., 2012). Our results support a negative effect on appetite in bees, at least, during the initial phase of the immune response. Anorexia could represent an adaptive behavior for forager bees, since it could reduce the motivation to forage and thus decrease the probability of predation. It also reduces the contacts with other bees inside the colony, as it would diminish the events of waggle dance or trophallaxis, limiting colony contamination.

It has been previously reported that infection affects social interaction in honey bees (Richard et al., 2008). We found that LPS-injected bees spend less time performing social behaviors (Figure 5A). This withdrawal from social interaction could be an adaptive response that could help preventing disease transmission, as it has been widely reported for different social insects (reviewed in Cremer et al., 2007). We also investigated the response of saline-injected bees in front of a sick conspecific. As social insects can show responses varying from intensive care to removal from the group (Arathi et al., 2000; Ugelvig and Cremer, 2007), we aimed to discriminate between aggressive and collaborative responses. We measured the number of attack events from a bee to another and found no significant differences between groups. Moreover, both saline and LPS-injected bees exhibit a tendency to lower attack levels when interacting with a sick bee. Besides this tendency, the absence of a counterpart effect in the time spent engaged in aggressive behaviors suggests that honey bees are more prone to display collaborative behaviors in front of conspecific sickness than aggressive ones. Social contact with infected individuals has been shown to provide survival benefit to other group members later challenged with the same pathogen in ant colonies (Ugelvig and Cremer, 2007), a phenomenon called social prophylaxis. A similar effect of group facilitation of disease resistance was also found in termites, an unrelated social insect (Traniello et al., 2002). This mechanism could have evolved as a way to counteract the higher risk of disease transmission given by group living.

Honey bee colony members usually share food through trophallaxis (Free, 1956). It has been established that trophallaxis does not only have a role in feeding, but also in sharing information about the nutritional value of the resources (Wainselboim and Farina, 2000) and in establishing associations between scents and food sources (Gil and Farina, 2003). In our study, we found a higher number of proboscis extension events in LPS-injected bees (Figure 5C), a behavior normally associated with food begging (Wright et al., 2012). However, a few considerations from our experiments suggest that the proboscis extension in LPS-injected bees might have a different origin. First, bees had food *ad libitum* in their respective compartments; thus, they are all expected to be satiated during the experiment. Second, our experiments show that LPS-injected bees are actually less eager for food. Third, consistent with the interpretation that the proboscis extension does not represent food begging in the present case, we have not observed any case in which proboscis extension in LPS-injected bees elicited trophallaxis from their partners. Thus, we hypothesize that the proboscis extension events observed might be related to sick bees transmitting some kind of alert or warning signal to conspecifics rather than food begging.

Reduction of exploration, appetite loss and decreased time spent performing social behaviors are all adaptive behavioral changes well documented in mammals, and altogether known as sickness behavior. It is very interesting to find similar results in honey bees, since with a less complex nervous system but with a very wide repertoire of behaviors, bees could provide a very useful tool to study sickness behavior, and also the interaction between the nervous and the immune systems. In mammals, the molecular mechanisms that orchestrate sickness behavior during an infection are only partially elucidated. Cytokines can reach the mammalian brain or their expression can be induced in brain cells, and they can in turn affect neuronal function and behavior (Depino et al., 2004, 2011; Lucchina et al., 2010). In honey bees and other insects, this communication between the immune and the nervous systems has been only partially studied. Eicosanoids and octopamine have been proposed as the main mediators of this coordination. Inflammatory stimuli increase octopamine haemolymph levels in crickets (Adamo, 2010), and this amine can modulate different behaviors associated with appetitive stimuli (Scheiner et al., 2006). Eicosanoids are the main mediators of the insect immune response: they are responsible of fever (Stanley et al., 2009) and they mediate nodulation upon a bacterial infection (Bedick et al., 2001). However, there is no evidence showing whether octopamine and/or eicosanoids modulate sickness behavior in insects. We then propose that the experimental paradigm developed here can be used to study the molecular and biochemical pathways that mediate the brain response to immune challenge in insects.

In addition, the description of sickness behavior provided here might help recognize diagnostic behaviors for healthier bee managing. Colony collapse disorder (CCD) is a multifactorial phenomenon associated with the massive loss of individuals in honey bee colonies, which has caused important economic losses around the world in recent years. Causes are related to infections, use of pesticides and a combination of different colony stressors (Johnson, 2015; Kielmanowicz et al., 2015; Moritz and Erler, 2016). In the present study we provide an initial description of behavioral symptoms related to an immune challenge. Understanding how an infection changes the behavior of the honey bees at the individual level will be of relevance to understanding how these alterations are scaled up to the colony level affecting the survival of hives.

## Author contributions

All authors contributed to the conception and design of the work, the acquisition, analysis, and interpretation of data. All authors participated in drafting the manuscript and approved the final version.

## Funding

This work was supported by the Argentinean UBACyT grant Nr. 20020110200162.

### Conflict of interest statement

The authors declare that the research was conducted in the absence of any commercial or financial relationships that could be construed as a potential conflict of interest.
